# 食品中芳香族氨基酸的分离分析技术研究进展

**DOI:** 10.3724/SP.J.1123.2022.04011

**Published:** 2022-08-08

**Authors:** Chenhui LU, Yi ZHANG, Yujie SU, Wenlong WANG, Yongwei FENG

**Affiliations:** 1.江南大学食品学院, 食品科学与技术国家重点实验室, 分析食品安全学研究所, 食品安全国际合作联合实验室, 江苏 无锡 214122; 1. State Key Laboratory of Food Science and Technology, International Joint Laboratory on Food Safety, Institute of Analytical Food Safety, School of Food Science and Technology, Jiangnan University, Wuxi 214122, China; 2.国家市场监管技术创新中心(特殊食品), 无锡市食品安全检验检测中心, 江苏 无锡 214142; 2. Technology Innovation Center of Special Food for State Market Regulation, Wuxi Food Safety Inspection and Test Center, Wuxi 214142, China

**Keywords:** 芳香族氨基酸, 吸附, 脱除, 分离, 综述, aromatic amino acids, adsorption, removal, separation, review

## Abstract

苯丙酮尿症等代谢病患者因基因缺陷无法正常代谢食物中的芳香族氨基酸(aromatic amino acids, AAA),常规饮食可能会造成永久性生理损伤,低AAA肽是其重要的蛋白质等同物来源。AAA分离分析技术对低AAA肽的制备和检测至关重要。该领域研究者探索了多种高效的吸附、分离材料,从复杂的蛋白质水解液中选择性吸附脱除AAA以制备符合特定氨基酸限量的低AAA肽类食品,或者建立对AAA特异性提取富集的分离分析策略。该文分析了AAA的结构特点与理化性质,总结了近年来基于活性炭、树脂等吸附材料脱除AAA的技术进展,并从样品前处理、手性分离和吸附-传感3个维度综述了二维纳米材料、分子印迹、环糊精、金属有机骨架等材料在AAA分离分析中的应用。通过探讨各类技术的优缺点,为AAA吸附脱除和分离分析技术的进步提供支撑。

## Abstract (en)

Amino acids are important building blocks of proteins in the human body, which are involved in many metabolic pathways. Patients with metabolic diseases such as phenylketonuria, tyrosinemia, and hepatic encephalopathy are genetically defective and cannot metabolize aromatic amino acids (AAA) in food; hence, a regular diet may lead to permanent physiological damage. For this reason, it is necessary to restrict the intake of AAA in their daily diet by limiting natural protein intake, while ensuring normal intake of low protein foods and supplementation with low-AAA protein equivalents. Sources of low-AAA protein equivalents currently rely on free amino acid complex mixtures and low-AAA peptides (also known as high-Fischer-ratio peptides), which have better absorption availability and palatability. AAA separation and analysis techniques are essential for the preparation and detection of low-AAA peptides. Researchers in this field have explored a variety of efficient adsorption materials to selectively remove AAA from complex protein hydrolysates and thus prepare low-AAA peptide foods, or to establish analysis strategies for AAA. Covering more than 70 publications on AAA removal and separation in the last decade from Web of Science Core Collection and China National Knowledge Infrastructure, this review analyzes the structural characteristics and physicochemical properties of AAA, and summarizes the technological progress of AAA removal based on adsorbents such as activated carbon and resin. The applications of two-dimensional nanomaterials, molecular imprinting, cyclodextrins, and metal-organic frameworks in AAA adsorption and analysis from three dimensions, i. e., sample pretreatment, chiral separation and adsorption sensing, are also reviewed. The mainstream adsorbents for AAA removal, such as activated carbon, still suffer from poor specificity and cause environmental pollution during post-use treatment. Existing AAA separating materials show impressive selective adsorption capability in food samples and chiral mixtures as well as high sensitivity in adsorption sensing. The development of an efficient detection technology for AAA may help in detecting trace AAA in food and in evaluating chiral AAA adulteration in food samples. By exploring the advantages and disadvantages of each type of technology, we provide support for the advancement of the removal and analysis techniques for AAA.

芳香族氨基酸(aromatic amino acids, AAA)包括苯丙氨酸(phenylalanine, Phe)、酪氨酸(tyrosine, Tyr)和色氨酸(tryptophan, Trp),其中Phe和Trp为必需氨基酸。Phe在体内的代谢途径主要是在苯丙氨酸羟化酶(phenylalanine hydroxylase, PAH)和辅酶四氢生物蝶呤(tetrahydrobiopterin, BH_4_)的作用下转化为Tyr。当患者由于基因缺陷导致体内缺乏PAH或BH_4_时,Phe会经由另一条代谢途径——转氨基作用产生苯丙酮酸,此时患者尿液中含有大量苯丙酮酸,称为苯丙酮尿症(phenylketonuria, PKU)^[1]^。Tyr在体内正常代谢可以产生儿茶酚胺和黑色素,而当Tyr分解代谢途径中存在酶缺陷时,会导致患者血浆中Tyr水平明显升高,称为酪氨酸血症。根据产生缺陷的酶种类不同,酪氨酸血症分为Ⅰ型(肝-肾型酪氨酸血症)、Ⅱ型(眼-皮肤型酪氨酸血症)和Ⅲ型。Trp是一种生糖兼生酮的氨基酸,戊二酸血症患者的Trp代谢途径中因戊二酰辅酶A脱氢酶缺陷导致戊二酸代谢产物堆积。上述AAA代谢异常会导致患者多个系统受损,甚至死亡。因此,需要严格限制此类患者饮食中的AAA^[2]^。根据食品安全国家标准GB 29922-2013《特殊医学用途配方食品(特医食品)通则》,苯丙酮尿症专用的特医食品中Phe含量不应超过1.5 mg/g蛋白质(等同物);酪氨酸血症专用的特医食品中Phe、Tyr含量不应超过1.5 mg/g蛋白质(等同物);戊二酸血症Ⅰ型专用的特医食品中Trp含量不应超过8 mg/g蛋白质(等同物)^[3]^。制备上述特医食品的途径一种是蛋白质水解物脱除特定氨基酸,另一种是游离氨基酸复配。蛋白质水解体系复杂,选择性高效脱除特定氨基酸的同时需要尽可能减少其他氨基酸和肽类物质的损失。复配游离氨基酸一般来源于工业化生产,产品中很可能含有生理差异巨大甚至毒性的对映体。因此,AAA的脱除和分析对制备和评价AAA代谢疾病患者的特医食品十分必要。

本文分析了AAA的结构特点与理化性质,总结了近年来基于活性炭、树脂等吸附材料脱除AAA的技术进展,并从样品前处理、手性分离和吸附-传感3个维度综述了基于二维纳米材料、分子印迹、环糊精、金属有机骨架等材料分离分析AAA的应用,通过探讨各类技术的优缺点,为AAA的吸附脱除和分离分析方法的发展提供参考。

## 1 芳香族氨基酸的种类与性质

3种AAA的结构如图1所示,由于分子中带有共轭芳环,因此可以吸收260 nm或280 nm的紫外光,这是AAA区别于其他氨基酸的重要特点。

**图 1:**
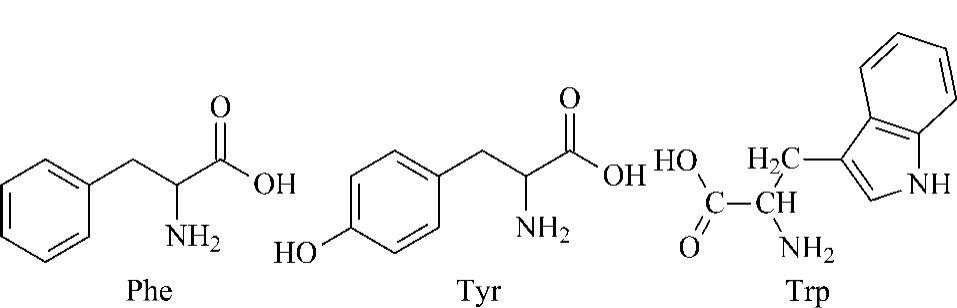
AAA的结构式

AAA的吸附与吸附剂性质(如表面电荷、孔结构、表面形貌和化学性质)、吸附环境(如pH、离子强度、溶剂组成和温度等)有关,也与AAA自身性质(如相对分子质量、分子结构、官能团、溶解度、极性)有关。分子间作用力在AAA的吸附过程中发挥着重要作用。

## 2 芳香族氨基酸的吸附脱除技术

蛋白质水解物类特医食品配料中的AAA脱除方法主要有超滤法、活性炭吸附法和树脂吸附法。超滤法分离效率高,但容易造成低相对分子质量营养成分(如游离氨基酸和寡肽)的损失^[2]^。吸附法具有不易影响生物活性、使用有机溶剂少、吸附过程pH变化小、设备简单且廉价、操作简便和安全性高等优点,被广泛运用在食品领域。以下介绍活性炭、树脂等材料在AAA吸附中的应用。

### 2.1 基于活性炭的芳香族氨基酸吸附脱除技术

活性炭由含碳的物料加工而成,是目前应用最广泛的吸附剂之一。活性炭对芳香族化合物的吸附能力大于脂肪族化合物,因此可以被用于AAA的特异性吸附。活性炭对AAA的吸附能力还受活性炭的理化性质和吸附环境的影响,孔径更小、比表面积更大的活性炭往往具有更强的吸附能力。与pH 6或pH 9相比,活性炭在pH 3的吸附体系中展现出对Phe更强的吸附能力,原因是氨基酸的等电点和活性炭的零电荷点在不同pH下会产生不同的静电作用^[4]^。Belhamdi等^[5]^以农业废弃物枣核为原料,通过化学活化法制备活性炭,发现活性炭对L-Trp的吸附为单层物理吸附,吸附能力取决于pH值和离子强度。

由于活性炭成本低、吸附速率快且对AAA具有一定的选择性,现已作为吸附剂广泛用于AAA的脱除、高*F*值(此处*F*值代表Fischer比率,指支链氨基酸与芳香族氨基酸的物质的量之比)的寡肽产品制备以及肝性脑病和PKU等代谢疾病患者的辅助治疗中。

Alves等^[6]^以玉米芯为原料,通过热处理使其转化为多孔活性炭用于Phe的吸附,其吸附机制是Phe的苯环与碳表面石墨烯环之间的*π-π*相互作用。Su等^[4]^对活性炭吸附Phe的机制进行了研究,发现吸附过程中疏水相互作用占主导地位。如图2所示,活性炭上的石墨环与Phe的苯环发生疏水相互作用,活性炭上的羟基和羧基与Phe上的极性分子和官能团发生反应,形成氢键和静电相互作用。

**图2:**
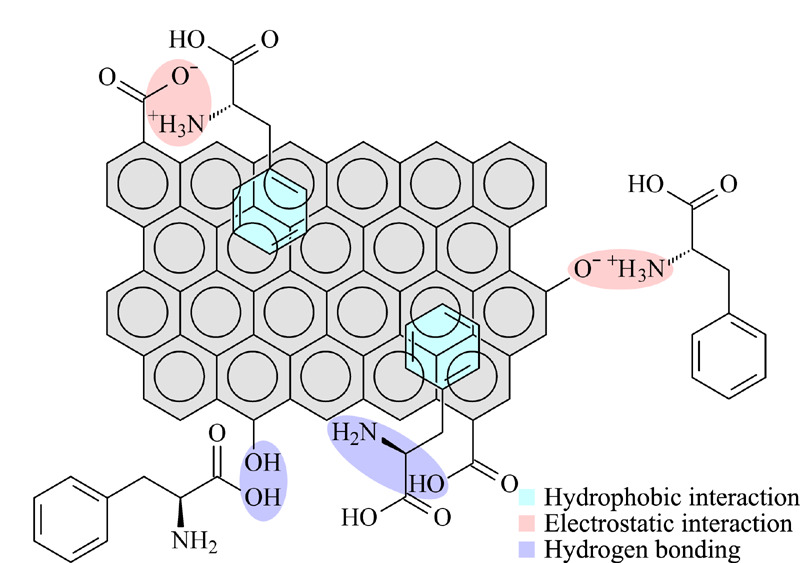
Phe与活性炭相互作用示意图

表1为文献报道的以活性炭为吸附剂制备的高*F*值寡肽产品。相比于其他技术,活性炭依旧存在吸附特异性差、难以回收、环境污染较大等问题,对活性炭纤维表面化学改性能适当改善吸附特异性差的问题。

**表1:** 以活性炭为吸附剂制备的高*F*值寡肽

Raw materials	Proteases	Adsorbents	Fischer ratio	Reference
Corn	alkaline protease, neutral protease	activated carbon	21.92	[7]
Corn	bacillus natto alkaline protease	modified activated carbon	21.80	[8]
Crude corn peptides	α-chymotrypsin, carboxypeptidase A	activated carbon (different type)	41.87	[9]
Tuna	pepsin, flavor protease	activated carbon (200 mesh)	30.33	[10]
Chlamys farreri	compound protease, flavor protease	activated carbon (200 mesh)	34.73	[11]
Antarctic krill	alkaline protease, flavor protease	activated carbon	21.12	[12]
Minced squid	pepsin, flavor protease	activated carbon (50 mesh)	/	[13,14]
Yeast	α-chymotrypsin, carboxypeptidase A	modified activated carbon	30.00-40.00	[15]
Soy protein	alkaline protease, flavor protease	activated carbon (different type)	18.90	[16]
Grass carp	pancreatin, papain	activated carbon (different type)	/	[17]
Pearl oyster	pancreatin	activated carbon	24.58	[18]
Goat milk	pepsin, flavor protease	activated carbon (200 mesh)	26.32	[19]

/ indicates no relevant data mentioned in the reference. Fischer ratio means the molar ratio of branched-chain amino acids to AAA.

### 2.2 基于树脂的芳香族氨基酸吸附脱除技术

#### 2.2.1 离子交换树脂

离子交换树脂是含有可交换离子的活性官能团的网状高分子化合物,分为大孔型和凝胶型。大孔型离子交换树脂具有较大的比表面积和吸附容量,吸附速度快,使用周期长,平均成本较低,且根据极性差异可以达到较好的选择性。因此,大孔型离子交换树脂是目前AAA吸附使用最多的树脂。AAA具有非极性结构,因此在进行AAA的吸附分离时通常选择非极性的大孔型离子交换树脂。Jiao等^[20]^选用弱极性的树脂XDA-200为吸附剂,发现L-Trp的吸附主要是基于*π-π*相互作用和疏水相互作用。在低浓度下,AAA与聚苯乙烯树脂基质之间存在非离子相互作用,其能够增强AAA在离子交换树脂上的吸附。

除与树脂本身的化学结构有关外,AAA的吸附效果还受吸附环境、吸附流速等因素的影响。随着离子强度的增加,AAA的吸附量随之增加。当料液初始pH值介于3.5~4.5时,对平衡吸附量影响不大,但pH≤2.0时则会降低AAA在树脂上的吸附量。张婷婷等^[21]^发现随着温度升高,001×7阳离子交换树脂对L-Trp的吸附率逐渐升高,但温度过高时,L-Trp的侧链与树脂的疏水相互作用被破坏,吸附量下降。

使用蛋白质水解物作为原料吸附脱除AAA制备特殊食品的过程中,水解液中的AAA可能以游离态和结合态两种形式存在。因此,吸附剂对蛋白质水解物中AAA的吸附能力需要进一步评估。吸附树脂对游离氨基酸有一定的选择性,但支链氨基酸也易被吸附,氨基酸分子的极性、分子结构和分子体积对吸附能力均有一定影响。赵谋明等^[22]^使用4种吸附树脂研究其对草鱼蛋白质水解物的吸附性能,发现比表面积大、孔径小的XDA-200具有更大的吸附量。Bu等^[23]^使用D101大孔树脂柱从乳清蛋白质水解物中去除Phe,吸附后水解物中Phe含量为(1.38±0.11) mg/g蛋白质等同物,且600 Da以下的肽段比例上升,测得水解物的苦涩味强度有所增加,但芳香活性化合物如美拉德反应和脂质氧化产物在吸附后减少。

#### 2.2.2 壳聚糖树脂

壳聚糖分子中的氨基、羟基等活性基团能够赋予其较强的吸附能力,交联和接枝功能性基团可以实现壳聚糖的选择性吸附。Zhang等^[24]^使用戊二醛作为交联剂,使用Phe修饰交联后的壳聚糖树脂,该材料具有从混合氨基酸溶液中特异性吸附AAA的能力,推测*π-π*疏水相互作用和静电相互作用在吸附过程中起主导作用。Jiang等^[25]^采用苯乙胺修饰壳聚糖树脂,实现了对AAA的选择性吸附,材料对3种AAA的吸附能力依次为Phe>Tyr>Trp。苯乙胺的苯环与Phe的苯环匹配度高能产生较强的疏水相互作用,而Tyr中的酚羟基会增大空间位阻,Trp中吲哚环的电子分布与Phe中的苯环不同,因而导致疏水相互作用降低。

### 2.3 其他潜在脱除方法

二氧化钛对AAA的亲和力优于脂肪族氨基酸,吸附作用由疏水相互作用主导^[26]^。AAA在锐钛矿(TiO_2_)纳米颗粒模型表面的吸附稳定性顺序为Tyr>Phe>Trp^[27]^。AAA结合亲水基团后,在TiO_2_表面的附着能力显著增加^[26,28]^。在GB 2760-2014《食品安全国家标准 食品添加剂使用标准》中,二氧化钛被允许作为着色剂用于糖果蜜饯、果酱、可可制品、固体饮料等食品类别^[29]^,可见其毒性较低,有望以吸附剂形式应用于食品工业。

此外,接枝*β*-环糊精(*β*-CD)的磁性碳纳米管可通过外加磁场吸附实现Trp的高效分离^[30]^。分子印迹技术可以提高AAA的分离选择性,进一步结合磁性纳米材料可以提高分子印迹聚合物在料液中的分离性能,有望实现工业连续化生产^[31,32]^。基于碳水化合物的反相胶束系统对AAA也有较好的包合效果,目前已经有两种新型Gemini表面活性剂用于AAA的吸附,AAA以Phe>Tyr>Trp的顺序被包封,且AAA的包封量随着表面活性剂疏水链长度的增大而增加^[33,34]^。沸石、固定化DNA膜和金属锗对AAA也有良好的吸附能力,其在食品体系中的适用性及其残留毒性有待评估^[35⇓-37]^。

## 3 芳香族氨基酸的分离分析技术

分离技术对食品中AAA的分析有重要作用。柱后衍生阳离子交换色谱法、柱前衍生高效液相色谱法以及高效阴离子交换色谱-积分脉冲安培检测法等均可用于氨基酸的分离分析^[38]^。但上述方法对AAA与对其他氨基酸无本质差异,在柱前并未对AAA做特异性分离富集。表2总结了部分材料在AAA特异性分离分析中的应用,以下将从样品前处理、手性分离和吸附-传感3个维度探讨基于选择性吸附的AAA分离分析技术。

**表2:** 部分材料在AAA特异性分离分析中的应用

Application	Target	Sample	Adsorbent	Analytical method	Reference
Sample	L-AAA	protein hydrolysate	MOFs	HPLC	[39]
pretreatment	AAA	lily	MWCNTs and MOFs composite	HPLC	[40]
	L-Trp	black sesame seed	GO@SiO_2_	HPLC	[41]
	AAA	AAA aqueous solution	ZIF-8@β-CDPs	/	[42]
Chiral	L-Phe	D,L-Phe; D,L-Trp	L-Phe-imprinted cryogel cartridge	FPLC	[43]
separation	L-AAA	D,L-AAA mixture	surface functionalized magnetic nanoparticles	HPLC	[44]
	D-Phe	D,L-Phe mixture	D,L-Ala-CD/SiO_2_	absorption spectroscopy	[45]
Adsorption-	L-Phe	artificial plasma	L-Phe imprinted SPR sensor	SPR	[46]
sensing	Phe, Tyr	standard solution	Ni@CNFs@Au	SERS	[47]
	Phe	AAA aqueous solution	pyrene@2βCD	absorption spectroscopy	[48]
	L-AAA	milk	bi-enzyme nanocomposite film-based biosensor	amperometric measurements	[49]

/ indicates no relevant data mentioned in the reference. MOFs: metal-organic frameworks; MWCNTs: multi-walled carbon nanotubes; GO: graphene oxide; ZIF-8: zeolitic imidazolate framework-8; *β*-CDPs: *β*-cyclodextrin polymer microspheres; CNFs: carbon nanofibers; FPLC: fast protein liquid chromatography; SPR: surface plasmon resonance; SERS: surface-enhanced raman scattering.

### 3.1 样品前处理

一些无机纳米材料和有机聚合物表现出了较强的AAA富集能力,被应用在AAA分析的样品前处理环节。Niu等^[41]^开发了一种以氧化石墨烯复合SiO_2_的纳米材料作为吸附剂,用于分析检测黑芝麻中的L-Trp。Keskinates等^[50]^制备了新型聚丙烯腈(PAN)和聚甲基丙烯酸甲酯(PMMA)纳米纤维基杯芳烃四酯单元,并发现其对AAA具有良好的结合能力。

金属有机骨架(metal organic frameworks, MOFs)具有孔隙率高、比表面积大、孔径均匀可调等优点,对AAA的吸附优于脂肪氨基酸,其吸附过程为吸热反应,机理涉及静电作用、氢键和*π*-*π*作用等^[51,52]^。陆思嘉等^[51]^使用溶剂热法制备了MOFs HKUST-1,对Tyr、Phe和Trp的饱和吸附量分别达到248.65、143.67、140.09 mg/g。Jonckheere等^[39]^选用Zr-MOF MIL-140C (Zr-4,4'-联苯二羧酸盐 MIL-140C)对水溶液和小麦秸秆蛋白质水解物溶液进行AAA吸附实验,发现与L-Phe苯基侧基相比,L-Trp因具有较大的吲哚基而更易与MIL-140C的疏水部分形成氢键,促进L-Trp的吸附,并与在水解物溶液中带负电荷的氨基酸发生共吸附。Li等^[40]^将MOFs与磁性碳纳米管结合制备了Fe_3_O_4_-MWCNTs-OH@poly-ZIF67,该材料对AAA表现出优异的选择性和吸附亲和力,可用于百合中3种AAA的选择性测定。

### 3.2 手性分离

对映体具有相同的化学和物理性质,但生物学性质和药理活性以及毒理学效应常存在明显的差异。例如,L-Phe广泛应用于制药和食品工业中的蛋白质生物合成,而D-Phe被认为可以抑制分解脑啡肽的酶活性,使其具有潜在的镇痛作用。因此,氨基酸对映体的分离分析在特医食品制备领域具有重要意义,而AAA手性识别材料的开发和应用是其关键技术。

环糊精是一种环状低聚糖,独特的环状空间结构形成了亲水外壳和疏水内腔,具有选择性包裹疏水性分子的能力。刚性*β*-CD聚合物树脂对Phe和Tyr有较强的吸附能力,其尺寸、刚性适中,热稳定性和机械稳定性较好,可用于固定床吸附和催化。李英杰等^[42]^采用原位生长法制备了ZIF-8@*β*-CDPs金属骨架复合微球,用于手性AAA的吸附。具有疏水结构和咪唑环的ZIF-8可与AAA的苯环相互作用,通过改变AAA的手性微环境使其更易吸附L-Phe。

磁性二氧化硅纳米粒子表面接枝羧甲基-*β*-CD,基于磁作用力可以实现AAA的手性分离^[44,53]^。对映体的疏水部分渗透到CD的疏水腔中形成包合物,AAA分子手性中心的氨基与吸附剂分子中的仲羟基形成氢键,促进了L-对映体的选择性吸附^[54]^。使用乙酸纤维素和海藻酸钠与*β*-CD共混合可制备用于Phe和Trp手性拆分的膜,该膜具有较高的热稳定性,对Trp和Phe对映体的最大分离率分别达到9.0%和10.9%^[55]^。使用*β*-CD键合二氧化硅,再使用上述材料和丙氨酸两种对映体分别衍生,以上3种材料对D-Phe的吸附能力明显高于L-Phe,对两者的拆分因子高达3.48,且具有良好的循环再生能力^[45]^。

丙烯酰CD和*N*,*N*'-亚甲基双丙烯酰胺与乙烯基化硅胶共聚可以增强材料的机械强度,可用于高效液相色谱固定相对Phe进行手性分离^[56]^。吴丽仙等^[57]^以D,L-Trp为模板分子,*β*-CD及其衍生物为单体,通过不同印迹体系和合成方法制备了一系列分子印迹聚合物,筛选出了能够在6种AAA的复杂体系中特异性拆分D,L-Trp的分子印迹聚合物,其拆分因子为1.477。利用自制的手性功能单体合成分子印迹聚合物或分子印迹膜可以实现对L-Phe的手性拆分^[58⇓-60]^。

基于分子印迹的冷冻凝胶柱可以通过两性离子使凝胶表面形成水化层,实现针对L-Phe的高效手性分离^[43]^。使用硅胶60F-254高效薄层色谱(HPTLC)板可分离D,L-Phe和L-Tyr,有机溶剂使用较少^[61]^。Lomenova等^[62]^使用高效液相色谱法对实际样品中的Phe进行分离和检测,发现在膳食补充剂样品中能够检测到两种对映体形式,在能量饮料样品中只检测到L-Phe,对两种对映体的检出限均为0.1 g/mL。毛细管电泳在反电渗模式下通过配体交换毛细管电泳法可以在较短时间内实现AAA的对映体分离^[63]^。

### 3.3 吸附-传感

表面增强拉曼散射(surface enhanced raman scattering, SERS)通过将分子吸附在金属纳米粒子的表面以增强分子的拉曼散射信号,这一技术可以用来识别传感AAA信号。与脂肪族氨基酸相比,Phe的特殊取代基团是苄基。如图3所示,在与金属原子(Cu、Au、Pt)发生吸附时,Phe的羧基端结合在金属原子的顶部位点上,苄基以平坦的构象吸附在金属原子上,促进*π*电子与底物之间的相互作用,降低结合自由能,维持吸附几何结构的稳定性^[64]^。SERS光谱可以鉴定出L-Phe和L-Trp上苯环和吲哚环的特征拉曼谱线,且拉曼信号强度与物质浓度具有良好的相关性^[65]^。Wu等^[47]^将Au沉积在碳纳米纤维包裹的磁性镍纳米颗粒上用于对AAA进行磁分离和SERS分析,Phe和Tyr的检出限分别为1×10^-11^mol/L和1×10^-13^mol/L。

**图3:**
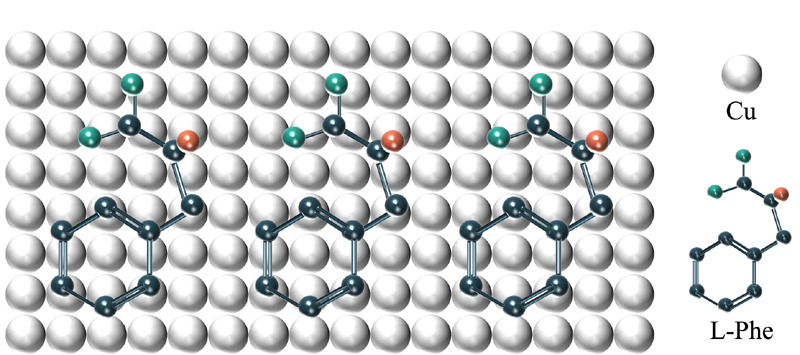
Phe与铜原子发生吸附作用的示意图

碳纳米管(carbon nanotubes, CNTs)结构类似卷曲石墨烯,带负电的羧基与带正电的氨基从管表面伸向水溶液中,既能提供静电斥力防止CNTs团聚,又可以提供氨基酸吸附位点^[66]^。相比于其他氨基酸,AAA对CNTs有更强的亲和力,两者形成的聚集体较为稳定^[67,68]^。相比于扶手形碳纳米管,锯齿形碳纳米管对Phe分子具有更大的结合能^[69]^。对单壁碳纳米管的氧化处理有利于氨基酸在纳米管上的吸附^[70]^,侧壁改性可以改变CNTs表面的理化性质,使其高效手性识别AAA。Wang等^[49]^将辣根过氧化物酶和L-氨基酸氧化酶固定在多壁碳纳米管修饰的玻碳电极表面制备成双酶纳米复合膜生物传感器,用于分析牛奶等复杂样品中的AAA,具有较高的回收率。

石墨或氧化石墨烯与Trp形成的复合物具有高稳定性,而石墨烯与Tyr的结合更倾向于平行取向;氧化石墨烯或石墨烯与Phe和Trp的相互作用主要表现为物理和化学吸附共同作用,与Tyr则表现出更强烈的化学吸附^[71⇓-73]^。石墨炔对AAA的吸附很敏感,在吸附过程中主要表现为化学吸附,有望开发为AAA生物传感器^[74]^。Hussain等^[75]^研究了AAA在二维硅烯和锗烯片上的结合原理,发现AAA通过物理吸附和化学吸附结合到两种薄片上,且AAA的引入改变了硅烯和锗烯纳米片的电子特性,有望用于AAA的检测。Song等^[76]^发现,3种AAA可以通过物理吸附作用吸附在MoS_2_的表面,AAA的苯环可与MoS_2_表面的硫原子之间产生平行相互作用。在Au修饰的MoS_2_表面,AAA通过与Au原子的共价连接及其与MoS_2_的非共价作用形成吸附,吸附能力表现为Trp>Tyr>Phe。碳纳米管、石墨烯等二维材料目前尚未被批准应用于食品加工工业,但其有可能在AAA分离分析中发挥巨大作用^[77]^。

微晶天平是一种新型的、极为灵敏的检测技术,在AAA吸附-传感领域有初步的应用。Mirmohseni等^[78]^在石英晶体电极上涂布分子印迹聚合物,可用于石英晶体纳米天平技术识别检测Phe。Cimen等^[46]^用紫外线聚合法将L-Phe分子印迹聚合物薄膜合成到表面等离子共振(SPR)芯片上,用于L-Phe的SPR传感检测。Titov等^[48]^制备了一种基于超分子芘-2-CD复合物的温度依赖型荧光传感器,可用于Trp和Phe的电子吸收光谱的检测。纤维素纳米晶体具有独特的光学性能,但Lombardo发现单个AAA不会吸附到纤维素纳米晶体上,与纤维素纳米晶体的结合可能需要氨基酸数量更多的序列^[79]^。

## 4 结论与展望

AAA选择性吸附技术在特医食品加工和检测领域都有至关重要的作用。尽管目前发现对AAA具有选择性吸附能力的材料种类丰富,但实际应用于食品原料中AAA吸附脱除的材料仍以活性炭和大孔树脂为主。这两种方法均存在特异性差的问题,前者还有一定的环境污染风险。通过进一步评估新型吸附材料的安全性,未来可以开发更多绿色环保、特异高效的吸附方法以改进AAA的吸附技术。此外,二维纳米材料、分子印迹技术和金属有机骨架等材料已初步运用于AAA分离分析,有望实现更高特异性和更高灵敏度的AAA分析检测。
